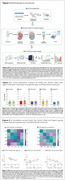# Early neural dysfunction in embryos of an autosomal dominant Alzheimer's Disease rat model

**DOI:** 10.1002/alz70855_106198

**Published:** 2025-12-24

**Authors:** André Nunes Mensch, Giovanna Carello‐Collar, Vanessa Gomes Ramos, Maria Luiza Fernandes Dahlem, Christian Limberger, Gabriela Lazzarotto, Débora Guerini de Souza, Diogo O. Souza, Eduardo R. Zimmer

**Affiliations:** ^1^ Universidade Federal do Rio Grande do Sul, Porto Alegre, Rio Grande do Sul, Brazil; ^2^ Universidade Federal do Rio Grande do Sul, Porto Alegre, RS, Brazil; ^3^ Brain Institute of Rio Grande Do Sul, PUCRS, Porto Alegre, RS, Brazil

## Abstract

**Background:**

Alterations in neuronal plasticity, excitatory/inhibitory imbalance, and neuroinflammation mediated by glial activation have been observed in Alzheimer's disease (AD) patients. Intriguingly, children and adolescents carrying an autosomal dominant Alzheimer's disease (ADAD) mutation present neuronal connectivity alterations, suggesting an early neural imbalance in AD. However, it remains unclear at what stage of development the dysfunction in the interplay among inhibitory neurons and glial cells emerge. Thus, we aimed to investigate the expression of genes related to inhibitory interneurons, astrocytes, and microglia during neurodevelopment in an ADAD rat model.

**Method:**

We collected brain tissue from wild‐type (WT) and TgF344‐AD (TG) embryos (e) at gestational day 13.5. Embryonic tissue was processed for DNA extraction and genotyping. The brain from paired embryos (WTe and TGe), derived from five WT and five TG progenitors, was used for complementary DNA synthesis. We quantified the gene expression of parvalbumin (*Pvalb*), somatostatin (*Sst)*, solute carrier family 1 member 3 (*Slc1a3*), glial fibrillary acidic protein (*Gfap*), and integrin subunit alpha M (*Itgam*) using RT‐qPCR (2^‐ΔΔCt^ method; Figure 1). Statistical comparisons between WTe and TGe were conducted with Student's t‐test. Gene expressions were correlated across groups and presented as Pearson Correlation matrices. Linear regressions examined the association between gene pairs (*p*‐value < 0.05).

**Result:**

No significant differences were observed for *Pvalb*, *Sst*, *Slc1a3*, *Gfap*, and *Itgam* expression between WTe and TGe (Student's t test, *p*‐values = 0.7594, 0.4359, 0.9705, 0.9833, and 0.1215, respectively; Figures 2A‐E). Significant correlation was observed between WTe *Pvalb* and *Itgam*, *Sst* and *Slc1a3*, and *Slc1a3* and *Gfap* gene expression (*p*‐value = 0.047, 0.025, and 0.014, Pearson Correlation Coefficient; Figure 3A, and linear regression; Figures 3Aa, 3Ab, and 3Ac, respectively). Interestingly, no significant correlation was observed for TGe (Figure 3B).

**Conclusion:**

Here, we show that the correlations identified in WTe were not detected in ADAD embryos, indicating early connectivity alterations and potentially future synaptic dysfunction. Further analyses should be conducted at advanced gestational days to better understand the effects of ADAD mutations on the interplay between inhibitory neurons and microglia during neurodevelopment.